# Integrated Genomic and Gene Expression Profiling Identifies Two Major Genomic Circuits in Urothelial Carcinoma

**DOI:** 10.1371/journal.pone.0038863

**Published:** 2012-06-07

**Authors:** David Lindgren, Gottfrid Sjödahl, Martin Lauss, Johan Staaf, Gunilla Chebil, Kristina Lövgren, Sigurdur Gudjonsson, Fredrik Liedberg, Oliver Patschan, Wiking Månsson, Mårten Fernö, Mattias Höglund

**Affiliations:** 1 Department of Molecular Pathology, Lund University, Malmö, Sweden; 2 Canceromics Branch, Department of Oncology, Lund University, Lund, Sweden; 3 Department of Oncology, Lund University, Skåne University Hospital, Lund, Sweden; 4 Department of Urology, Skåne University Hospital, Malmö, Sweden; Deutsches Krebsforschungszentrum, Germany

## Abstract

Similar to other malignancies, urothelial carcinoma (UC) is characterized by specific recurrent chromosomal aberrations and gene mutations. However, the interconnection between specific genomic alterations, and how patterns of chromosomal alterations adhere to different molecular subgroups of UC, is less clear. We applied tiling resolution array CGH to 146 cases of UC and identified a number of regions harboring recurrent focal genomic amplifications and deletions. Several potential oncogenes were included in the amplified regions, including known oncogenes like *E2F3*, *CCND1*, and *CCNE1*, as well as new candidate genes, such as *SETDB1* (1q21), and *BCL2L1* (20q11). We next combined genome profiling with global gene expression, gene mutation, and protein expression data and identified two major genomic circuits operating in urothelial carcinoma. The first circuit was characterized by *FGFR3* alterations, overexpression of *CCND1*, and 9q and *CDKN2A* deletions. The second circuit was defined by *E3F3* amplifications and *RB1* deletions, as well as gains of 5p, deletions at *PTEN* and 2q36, 16q, 20q, and elevated *CDKN2A* levels. *TP53/MDM2* alterations were common for advanced tumors within the two circuits. Our data also suggest a possible RAS/RAF circuit. The tumors with worst prognosis showed a gene expression profile that indicated a keratinized phenotype. Taken together, our integrative approach revealed at least two separate networks of genomic alterations linked to the molecular diversity seen in UC, and that these circuits may reflect distinct pathways of tumor development.

## Introduction

Cytogenetic and traditional comparative genomic hybridization (CGH) studies of urothelial carcinoma (UC) have revealed several recurring chromosomal alterations [1], [2], [3]. Particularly frequent are losses of chromosome arms 9p and 9q, amplifications at 6p22, and deletions of the *RB1* tumor suppressor gene on chromosome 13q [4], [5]. Array-based CGH (aCGH) studies have been instrumental in delineating genomic regions that are targeted by amplifications and deletions. In an early study, Veltman et al. identified several candidate oncogenes in recurrent high-level amplifications, e.g., *E2F3* (6p22), *CCND1* (11q13), and *CCNE1* (19q13) [6]. Subsequent aCGH studies have corroborated these results and identified several additional recurrent genomic aberrations, of which, amplifications of the TP53 antagonist MDM2, and homozygous deletions at 9p21 (*CDKN2A*), at 10q23 (*PTEN*), and at 9q33, a region covering the *DBC1* gene, are some examples [7], [8], [9]. Apart from chromosomal changes, a number of recurring point mutations have been reported, of which the most common are activating mutations of the *FGFR3* and *PIK3CA* genes, as well as inactivating mutations of *TP53* and genes involved in chromatin remodeling [10], [11]. The accumulated data have shown that *FGFR3* mutations are characteristic for low grade and low stage tumors [12] whereas *TP53* mutations are characteristic for invasive tumors. This has lead to the suggestion that UC develop through at least two molecular pathways, one related to *FGFR3* and one to *TP53* [13], [14]. In the present investigation we use tiling resolution aCGH on a series of 146 UCs and describe a number of putative target genes for recurrent genomic alterations. We further address the relationship between impaired TP53 activity, chromosomal instability and level of genomic rearrangement. Finally, by integration of genomic and mutation data with gene expression profiling we delineate two major genomic circuits of central importance for UC development.

## Results

### Recurrent genomic alterations in urothelial carcinoma

**Figure 1:**
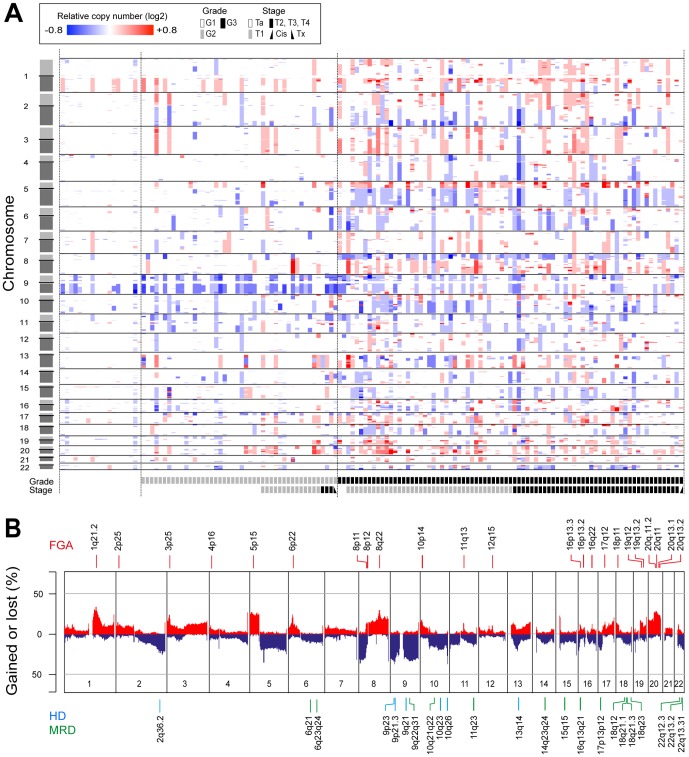
DNA copy number alterations in 146 bladder tumors. **A**) Whole genome heatmap representing relative copy number profiles of the samples. Segments of gains or deletions are color-coded according their relative log2 copy number ratios. **B**) DNA copy number frequency plot of gains (red) and losses (blue). Above: Recurrent high-level focal amplifications (FGA; red) are indicated by red bars and labeled according to their cytogenetic localization. Below: Recurrent homozygous deleted regions (HD; blue) and recurrent minimal regions of deletions (MRD; green) labeled according to their respective cytogenetic localization.

We performed genome wide DNA copy number profiling of 146 cases of UCs using tiling-resolution BAC arrays (Figure 1A). Genomic regions particularly affected by copy number gains were observed on chromosome arms 1q, 3p, 3q, 5p, 6p, 8q, 18p, 20p, and 20q, whereas deletions were common on 2q, 5q, 8p, 9p, 9q, 10q, 11p, 13q, 17p, and 22q (Figure 1B). In line with previous reports, a strong association between increasing numbers of copy number alterations and pathological stage and grade was observed (Figure 1A and Figure S2). Grade 1 tumors carried few alterations: primarily deletions of 9q, but also occasional deletions of 9p and gains of 1q whereas G2 tumors showed a slightly wider spectrum of aberrations. In particular, the frequency of *CDKN2A* deletions was increased in G2 compared to G1; 48% vs. 16% (Figure S2A). A major transition with respect to genomic alterations was observed between G2 and G3 tumors as G3 tumors harbored markedly higher numbers of genomic alterations (Figs. 1A and S2A).

We identified 31 recurrent regions of focal genomic amplification (FGA), 16 minimal regions of deletion (MRD), and 7 regions of recurrent homozygous deletions (HD) (Figure 1B and Table S2). The most frequent MRDs were seen on chromosome 9 (9p21, and 9q22-q31), followed by 10q23-q25, and 17p13.2. Known tumor suppressor genes, *e.g.*, *CDKN2A* (9p21.3), *PTEN* (10q23), and *RB1* (13q14) were located within HD regions, as well as genes with potential tumor suppressor properties, *e.g.*, *CUL3* (2q36.2) and *MGMT* (10q26). The most frequent regions of copy number gain were observed at 1q23 (34%), chromosome arm 5p (27%), 8q22 (29%), and 20q12 (28%) and the three most common FGAs were observed at 6p22, 11q13, and 8q22. Apart from previously well-described oncogenes, *e.g.*, *E2F3* (6p22), *CCND1* (11q13), *CCNE1* (19q12), *MDM2* (12q15), *RAF1* (3p25) *FGFR3* (4p14), and *ERBB2* (17q12), a number of genes with potential roles in tumor development were located within FGAs, including *SETDB1* (1q21), *BCL2L1* (20q11), and members of the *YWHA* (14-3-3) gene family (Table S2). We further noticed that the paralogous genes *CCND1* and *CCNE1*, *ID1* and *ID2*, and *YWHAZ*, *YWHAB*, and *YWHAQ*, showed alternate amplification patterns resulting in combined frequencies of 16%, 8%, and 18% respectively.

**Figure 2:**
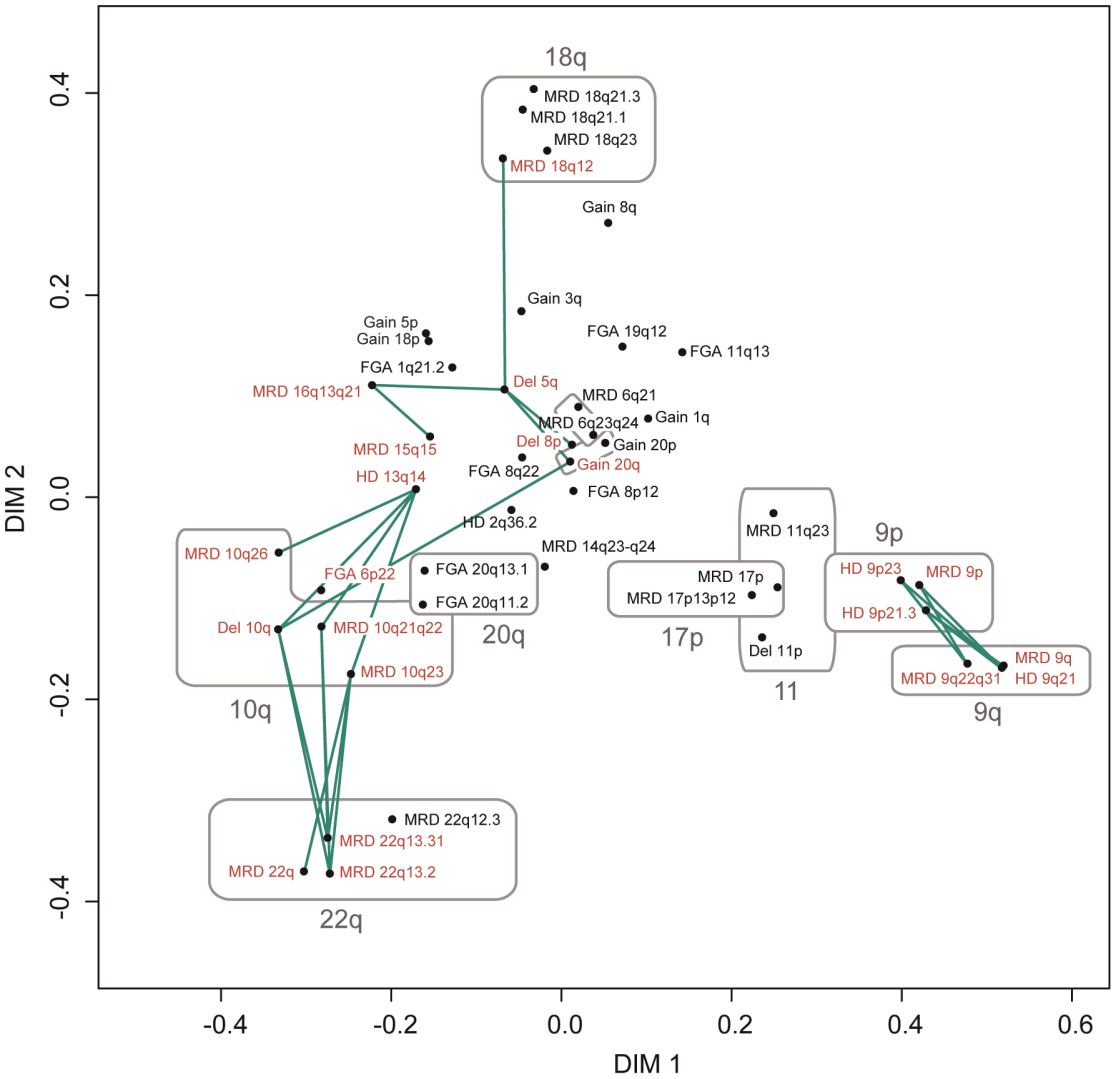
Associations between chromosomal aberrations visualized by MDS. Recurrent FGAs, HDs, and MRDs, as well as recurrent large chromosome arm deletions were included in the analysis. FGAs and HDs present in <5% of samples were excluded. Aberrations with significant positive associations, as determined by hypergeometric tests, are indicated in red and connected with green lines. Aberrations located to the same chromosomes are circled in gray for visualization purposes.

To visualize associations between genomic imbalances we performed multidimensional scaling (MDS) on all recurrent genomic alterations (Figure 2). Moreover, significant associations (Bonferroni corrected p<0.05, hypergeometric tests) were found between 19 combinations of aberrations (Figure 2). These analyses thus highlighted connections between different sets of genomic imbalances. For example, deletions on 9p, 9q, and 11p were connected, whereas 6p22 amplifications were associated with losses on 13q14, 10q, and 20q (Figure 2).

### Genomic complexity is associated with global gene expression patterns

**Figure 3:**
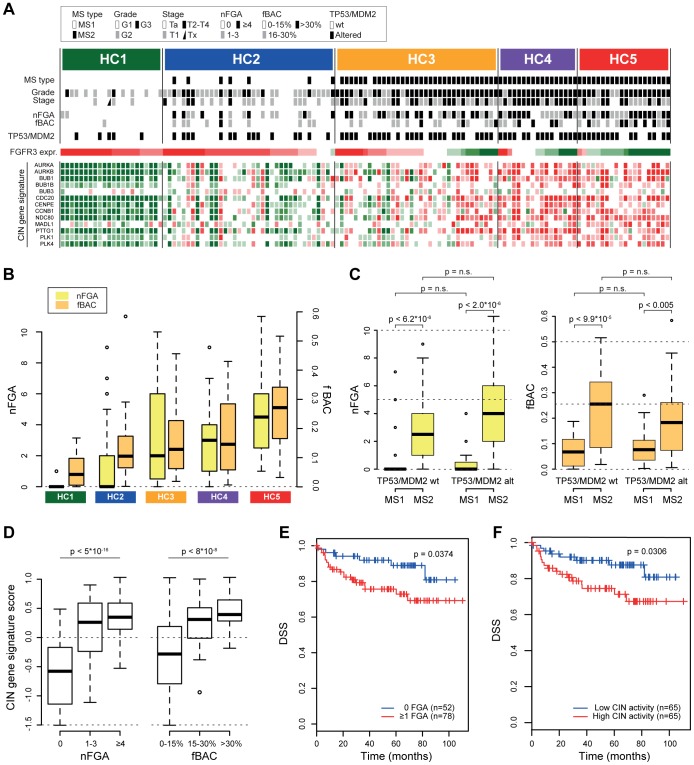
Genomic complexity is associated with UC gene expression subtypes. **A**) Hierarchical cluster analysis (HCA) on gene expression data segregated the tumors into five clusters, HC1 to HC5. Samples within each HCA group are ordered according to their relative FGFR3 expression (high expression left, low expression right). For each individual tumor, molecular signature (MS) type, pathological grade, stage, nFGA, fBAC, and TP53/MDM2 status is indicated. The relative expression levels for genes within the CIN signature are indicated by a heatmap below (green, low expression; red high expression). **B**) Boxplot illustrating the number of FGAs (nFGA) and frequency of genomic imbalances (fBAC) for samples within each HCA group. **C**) Boxplot of nFGA (left) and fBAC (right) for tumor samples when grouped on TP53/MDM2 status and MS type. P-values obtained by Wilcoxon statistics. n.s., not significant. **D**) Boxplot illustrating increased CIN score for samples with increased nFGAs (left) and fBAC (right). P-values obtained by ANOVA. **E** and **F**) Disease specific survival (DSS) analysis with tumors grouped according to nFGAs (0 vs ≥1 FGA) and CIN pathway score (above or below median), respectively.

We next aimed to investigate how genomic alterations are related with gene expression subgroups of UCs. We therefore stratified tumors into subgroups based on global gene expression data using hierarchical cluster analysis (HCA; Figure S3A). Two major clusters of tumors were identified, one dominated by G1/G2 tumors, split into two subgroups (HC1 and HC2), and a second, dominated by G3 tumors segregating into three subgroups (HC3, HC4, and HC5). As expected, the two largest HCA branches corresponded to the two main molecular subtypes (MS) previously described for bladder cancer [15]; MS1 tumors were confined to HC1 and HC2 whereas HC3, HC4, and HC5 were of the MS2 type (Figure 3A).

We then used two measures to estimate levels of genomic complexity: the total number of FGAs (nFGA) and the fraction of altered BAC clones (fBAC). Both parameters showed a strong association with the gene expression clusters (Figure 3A and 3B). In our cohort, 56% of the samples carried either non-synonymous mutations in *TP53*, or showed amplification or overexpression of *MDM2*, which targets TP53 for degradation (Figure S4A). We have previously shown that *TP53/MDM2* alterations are not strictly associated with genomic instability (15), and in the present, extended series of tumors, neither nFGA, nor fBAC, were significantly associated with *TP53/MDM2* status within each MS subtype (Figure 3C). When, however, these measures were compared between MS1 and MS2 tumors within *TP53/MDM2* altered and wild type cases separately, significant associations with MS subtype was observed (Figure 3C). Hence, genomic complexity shows a stronger association with MS type than with *TP53/MDM2* status. We then used a gene expression signature for chromosome instability (CIN) [16] and calculated a CIN gene expression score for each tumor. The CIN genes showed a clear association with HCA clusters (Figure 3A) and correlated significantly with both nFGA (r = 0.58) and with fBAC (r = 0.54). Moreover, a significant increase in CIN score was observed for tumors with FGAs and for tumors with increased fBAC (Figure 3D). However, no difference in scores was noted between cases with few or many FGAs, or between cases with medium or high fBAC. Importantly, both the CIN score and the presence of FGAs were associated with adverse patient survival (Figs. 3E and 3F).

### Molecular subgroups of UC are characterized by distinct genomic alterations

**Figure 4:**
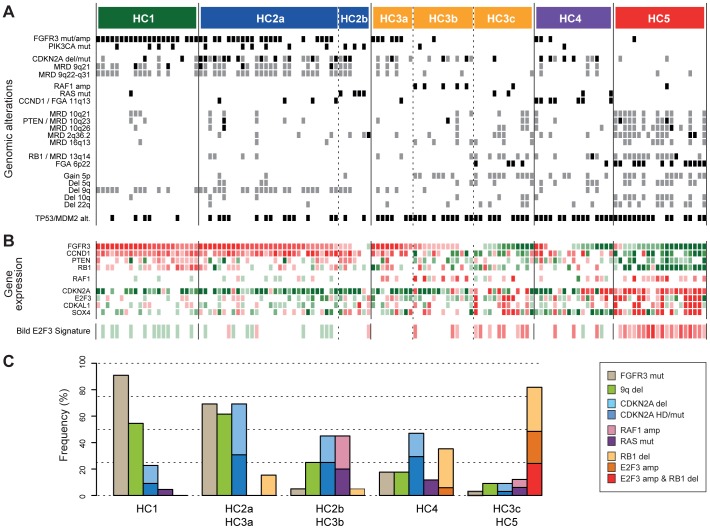
Integrated analysis of genomic alterations, gene mutations, and gene expression data. **A**) Recurrent genomic alterations with significant association to gene expression subtypes. Activating mutation of *FGFR3*, *PIK3CA*, and *RAS*, and inactivating mutations of *CDKN2A*, as well as amplifications of *FGFR3* and *RAF1*, and TP53/MDM2 status is also displayed. Within each HCA group, samples are ordered according to their relative *FGFR3* expression. Dashed vertical lines, which define subsets within each HCA group, are drawn with respect to the E2F3, RB1, 5p, RAF1, and RAS alterations pattern and FGFR3 expression. Amplifications and homozygous deletions are indicated in black. Gains and deletions are indicated in gray. Activating/inactivating mutations are indicated in black. **B**) Heatmap representing relative expression levels of selected genes and the Bild E2F3 signature [17]. Green, low expression; red high expression. **C**) Frequencies of selected genomic alterations in gene expression subgroups.

The results presented in Figure 2 suggested that two separate branches of interconnected genomic alterations may be present in UCs. To explore this finding further, we identified recurrent genomic aberrations that were significantly associated (Bonferroni corrected p<0.05, Fisher's tests) with the five gene expression subgroups shown in Figure 3A. Ten focal events (FGAs, HDs or MRDs) and six large chromosome arm alterations were significantly associated with specific HCA groups (Figure 4A). The most common chromosomal alteration in HC1 was deletions on whole or large segments of 9q (50%). HC2 tumors showed frequent losses on both 9p and 9q, and a high frequency (65%) of hemizygous/homozygous *CDKN2A* deletions. HC1 and HC2 also displayed a high prevalence of *FGFR3* and *PIK3CA* mutations (Figure 4A), and consistent high *FGFR3* and *CCND1* expression (Figure 4B). However, genomic amplifications of *CCND1* were associated with HC4 (7 of 17 HC4 cases; Figure 4A).

More than half (60%) of samples in HC5 harbored 6p22 amplifications. In addition, deletions of the *RB1* locus were frequent (65%) and accompanied by reduced *RB1* gene expression (Figs. 4A and 4B). A closer inspection revealed that three additional HC5 cases carried intra-chromosomal breakpoints surrounding the *RB1* locus (Figure S5). Thus, almost all tumors (95%) in HC5 harbored genomic alterations at 6p22 and/or 13q14, strongly indicative for a hyper-activation of the E2F pathway. This was further supported by increased expression of an E2F3 gene signature described by Bild et al. [17] (Figure 4B). Moreover, by organizing tumors within each HCA cluster according to *FGFR3* expression, a subset of HC3 tumors (HC3c) with low *FGFR3* expression that showed 6p22 amplifications and/or *RB1* losses in close to 70% of the cases, along with increased expression of the Bild E2F3 signature. This was contrasted by the HC2 group of tumors, characterized by *FGFR3* mutations and *CDKN2A* alterations, within which only 11% of the samples carried 6p22 and/or RB1 alterations (Figure S5). Interestingly, 6p22 and *RB1* alterations were associated with a relative overexpression of *CDKN2A* (Figs. 4B and S6A). This observation was further corroborated by an immunohistochemical analysis of p16 protein expression using tissue microarrays (TMA) comprising 119 of the samples. 6p22*/RB1* altered tumors showed significantly higher p16 levels than histologically normal urothelium and tumors without 6p22*/RB1* alterations (p<0.0001, t-test; Figs. S6B–6E).

Our data further suggested the presence of a specific *RAF1/RAS* related group of tumors as 5 out of 7 *RAF1* amplified tumors localized to a subset of HC3 tumors with moderate FGFR3 expression (HC3b) and the majority of *RAS* mutated tumors formed a subgroup within HC2 (HC2b). Only one tumor in these two subgroups harbored an *FGFR3* mutation and no overexpression of *FGFR3* was observed. In fact, *RAF1*/*RAS* alterations showed a significant negative association with *FGFR3* mutations (p<0.002, hypergeometric test) suggesting that these alterations may be complementary to each other.

### Two genomic circuits in urothelial carcinoma

**Figure 5:**
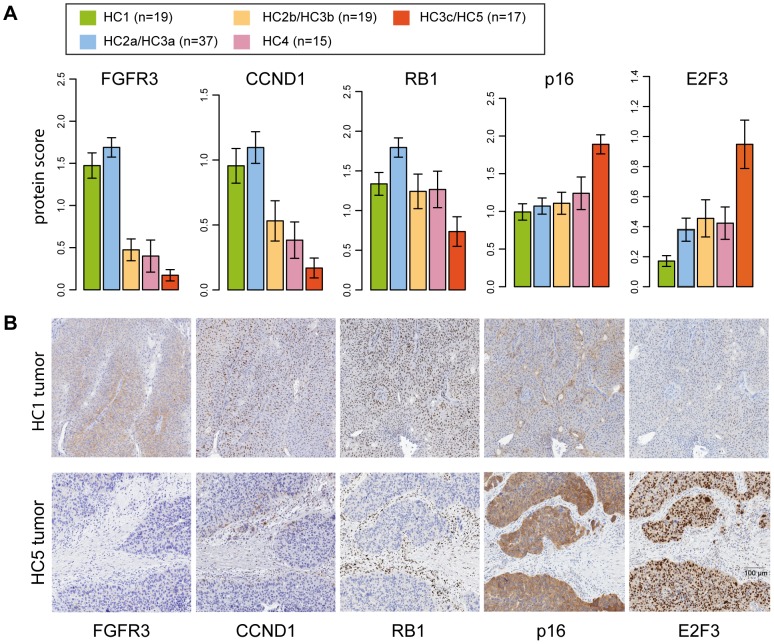
Validation of gene expression data by IHC on tissue microarray. **A**) Barplots summarizing tumor cell protein scores of selected proteins in tumors stratified according to the gene expression subtypes. Error bars represent ±SEM. **B**) IHC stainings of two representative HC1 (top) and HC5 samples (bottom).

The above analysis led us to refine the subgrouping of tumors into groups that share central features of gene expression, genomic alterations, and gene mutation data (Figure 4C). Two of the groups, HC1 and HC2a/HC3a, respectively, were highly enriched for *FGFR3* mutations, high *CCND1* expression, and 9q deletions. However, for HC2a/HC3a tumors the frequency of *CDKN2A* deletions was drastically increased, suggesting that acquisition of *CDKN2A* deletions occurs later during tumor progression than for example *FGFR3* mutations and 9q deletions. The tumors in HC3c and HC5 did, on the other hand, seem to depend on alternative genomic and genetic alterations as suggested by the high incidence of 6p22 amplifications and *RB1* deletions, and the near absence of both *FGFR3* mutations and *CDKN2A* deletions (Figure 4C). These observations were substantiated by immunohistochemical analysis of FGFR3, CCND1, p16, E2F3, and RB1 protein expression in 119 matched samples using TMA (Figure 5A). Protein expression of these markers for two representative samples of the HC1 and HC5 subgroups, respectively, is illustrated in Figure 5B.

**Figure 6:**
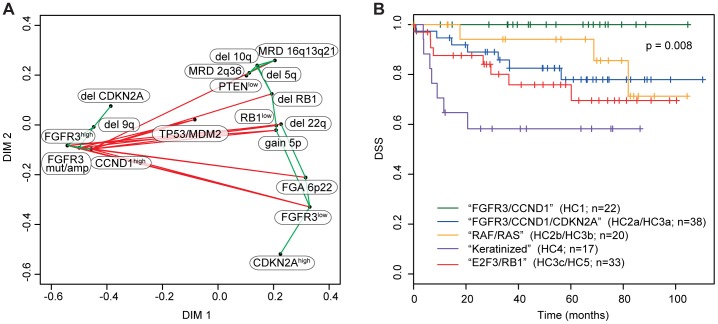
Genomic networks and survival analysis of genomic subtypes. **A**) MDS plot based on the subset of genomic alterations (Figure 4A) and categorized gene expression data (Figure S3B) that showed at least one instance of significant positive or negative association in a pair-wise hypergeometric tests. Green lines, significant positive association; red lines, significant negative association. **B**) Kaplan-Meier analysis of tumors grouped according to a combination of gene expression and genomic alteration patterns using disease specific survival (DSS) as endpoint.

The skewed distribution of genetic and genomic alterations between UC subgroups indicated the presence of at least two, possibly three, genomic circuits in UC. To test this more rigorously we performed pairwise hypergeometric tests between all aberrations that were associated with gene expression subclusters. Furthermore, to be able to include gene expression in this analysis, *FGFR3* expression were categorized in to high, intermediate, and low expression, and *CCND1*, *RB1*, *PTEN*, *CDKN2A*, and *RAF1* into low and high expression (Figure S4B). In Figure 6A the results are given in the form of a simplified network model. This analysis identified *FGFR3* mutations and elevated *FGFR3* expression, high *CCND1* expression, *CDKN2A* deletions, and deletion 9q as one circuit (the FGFR3/CCND1 circuit). The other circuit, the E2F3/RB1 circuit, was defined by 6p22 amplifications, deletions of *RB1*, *PTEN*, 5q, 2q36, 22q, and 16q, and gains of 5p, accompanied by reduced expression of *FGFR3*, *RB1*, and *PTEN*, and high expression of *CDKN2A*. The analysis also suggested the presence of a *RAF1*/*RAS* route, complementary to *FGFR3* mutations; however, this circuit did not attain statistical significance, possibly due the relative low frequency of these alterations.

We finally performed a survival analysis with the refined group definitions inferred in Figure 4C. No patients within the HC1 group succumbed to the disease during the time of follow up (Figure 6B). An adverse outcome was however observed for patients within the HC2a/HC3a group. Similarly, decreased survival rates were observed for the two groups enriched with *RAF1*/*RAS* and 6p22/*RB1* alterations. However, the worst outcome was noted for HC4 patients, which, except for a significant enrichment of *CCND1* amplifications and a high rate of *TP53*/*MDM2* alterations, were heterogeneous with respect to the setup of genomic alterations. Also, tumors within this group were less genomically complex than the HC5 subgroup (Figure 3B). We therefore used gene expression data to identify genes with significant upregulation in HC4 relative to the other gene expression clusters. The obtained gene signature was highly enriched for genes that indicate a keratinized phenotype, such as *KRT6A*, *KRT6B*, *KRT6C* and *KRT16*, as well as *SPRR1B*, *SPRR2A*, *SPRR2D*, and *SPRR2F*. Importantly, a histopathological reevaluation corroborated these findings: 7 of the 17 HC4 tumors showed signs of squamous differentiation. Thus, our data stress a subgroup of UCs with diverse setup of underlying genomic alterations, but with an expressional program connected to keratinization, that is associated with a highly unfavorable prognosis.

## Discussion

In this study, we performed an integrated analysis of genomic alterations, gene expression, and gene mutation data on a large series of urothelial carcinomas. To start with, we defined chromosomal boundaries for regions targeted by recurrent genomic alterations. In line with previous data, we observed recurrent amplifications (FGAs) at, *e.g.*, 6p22 (*E2F3*), 11q13 (*CCND1*), 8q22 (*YWHAZ*), 3p25 (*RAF1*), 4p16.3 (*FGFR3*), 12q15 (*MDM2*), and 17q12 (*ERBB2*), to mention some. We also identified several novel genes and chromosomal regions of possible importance for UC tumorigenesis. For example, a common region of amplification at 1q21.2 included the histone methyltransferase *SETDB1*, which has been implicated in the onset of melanomas [18], and the apoptotic regulator *BCL2L1* at 20q11 was also found recurrently amplified. Notably, we found several instances of amplifications of paralogous genes. For example, a large fraction (18%) of the tumors showed genomic amplification of either *YWHAZ*, *YWHAB*, or of *YWHAQ*. The *CDKN2A* locus was by far the most frequent homozygous deleted (HD) locus in our data. However, HDs were also observed for the well-known tumor suppressor genes *PTEN* and *RB1*. Interestingly, we observed HDs that involved the DNA methyltransferase *MGMT* (10q23) and the *CUL3* gene (2q36.2), that may be possible targets for the frequent deletions observed at 2q and 10q. The recurrent regions of deletion (MRDs) were typically wide, thus resulting in large numbers of potential target genes. For example, we were able to narrow down a region on 9q that, among others, included the *PTCH1* gene that previously has been postulated as a UC specific tumor suppressor gene [19]. However, this region also contained the XPA gene, important for DNA excision repair, and implicated in neoplastic transformation.

We next estimated the level of genomic complexity for the tumors based on chromosomal alterations (nFGA and fBAC), as well as indirectly using a published gene signature for chromosomal instability (CIN; 20). These genomic complexity measures were analyzed in relation to matched global gene expression profiling data and *TP53*/*MDM2* alteration status. Through this approach we could extend our observations from a previous study [15] in that the link between genomic complexity and the two intrinsic molecular subtypes of UC (MS1 and MS2) is stronger than what is observed between genomic complexity and *TP53*/*MDM2* alterations. That is, MS2 cases wild type for *TP53/MDM2* show as rearranged genomes as mutated cases. In contrast, MS1 cases, with or without *TP53/MDM2* alterations, show significantly lower levels of genomic complexity. Based on these findings we hypothesize that *TP53/MDM2* alterations have different biological meanings in MS1 and MS2 tumors, respectively. In MS1 cases, *TP53/MDM2* alterations may primarily function to promote protection from oncogenic stress signals [20]. The presence of these alterations in MS2 cases may, on the other hand, indicate selection of cells resistant to apoptotic signals associated with genomic instability. Therefore, *TP53/MDM2* alterations do not have to be causative for genomic instability *per se*. We also compared the CIN signature between tumors with increasing number genomic alterations. A major transition was observed between tumors with low numbers of aberrations as compared to tumors with intermediate levels. However, no significant difference was seen between the groups of intermediate as compared to high numbers of genomic alterations. Taken together, our findings indicate that UC may be categorized into tumors with simple and with complex genomes, *i.e.* into tumors that have passed through a period of genomic instability and tumor that have not, and that this distinction does not seem directly dependent on the presence of *TP53/MDM2* alterations.

The high prevalence of *FGFR3* mutations seen in non-invasive tumors is contrasted by infrequent mutations of this gene within the group of muscle invasive tumors. Moreover, invasive cancers often present without history of non-invasive disease. It has therefore been suggested that UCs may arise through at least two divergent molecular pathways: one associated with FGFR3 and the other with TP53 and RB1 alterations [13], [14]. However, details regarding cooperative alterations within these suggested pathways are far from clear. In order to elucidate these matters, we used matched global gene expression to cluster our samples into distinct molecular subgroups. We then used stringent statistical tests to identify genomic alterations that were associated with the obtained molecular phenotypes. Using this approach we could define two main genomic circuits in UC. Central alterations within these circuits were alterations of *FGFR3*/*CCND1* and *E2F3*/*RB1*, respectively, with several additional alterations linked to each of the two routes. Our analyses also suggested that *RAF1* and *RAS* alterations, which were found in a near mutual exclusive manner to *FGFR3* mutations, defined a possible third but less well established route.

In the first circuit, a strong link between FGFR3 hyperactivation and overexpression of CCND1 was noted, indicating that these tumors are receptor pathway driven through activation of the early G1 phase of the cell cycle. Moreover, tumors within this circuit segregated into two molecular phenotypes: both of which harbored frequent 9q deletions, 1q gains and *PIK3CA* mutations, but with *CDKN2A* deletions almost exclusively observed in the group with increased numbers of genomic alterations and a worse survival. We therefore suggest that *CDKN2A* inactivation occurs secondary to 9q deletions in the *FGFR3* circuit. In this scenario *CDKN2A* loss is coupled to tumor progression rather than initiation, perhaps by further promoting a CCND1 driven activation of the cell cycle G1 phase of the cell cycle.

Two major components of the second circuit were amplifications of the *E2F3* locus at 6p22 and reduced *RB1* expression. Our data thus corroborate the close link between 6p22 amplifications and *RB1* inactivation observed in UCs by Hurst et al. [21]. The importance of E2F3 induced gene expression was shown by consistent overexpression of the E2F3 Bild et al. gene signature in E2F3/RB1 associated cases. *E2F3* expression in combination with low *RB1* expression indicates that these tumors, in contrast to the FGFR3/CCND1 tumors, may be independent of the G1 restriction point and instead driven by the late G1 phase of the cell cycle. Additional changes within the E2F3/RB1 circuit included, among others, 10q deletions and 5p gains. Interestingly, reduced *PTEN* expression was specific for the E2F3/RB1 group in contrast to *PIK3CA* mutations that associated with the *FGFR3* circuit. In fact, a significantly lower *PTEN* expression was observed for tumors without *PIK3CA* mutation. Our data thus suggest that acquired alterations of *PIK3CA* and *PTEN* are complementary and may in fact be subtype specific in UC. This is in line with data from a recent study of transgenic mice that demonstrated that concurrent inactivation of *pten* and *tp53* in mouse bladder epithelium led to invasive tumor growth [22]. In most published studies, gains or amplifications on 5p have involved large segments and often the entire chromosome arm [4], [9], [14]. Consequently, no obvious 5p target genes have been identified. Irrespectively, the association of 5p with high grade and invasive tumors has been repeatedly reported and we show that 5p may be an integral part of the aggressive phenotype observed for tumors in this circuit.

The finding that the E2F3/RB1 circuit was specifically associated with low *FGFR3* expression indicates this circuit as essentially different from FGFR3/CCND1 tumors. Furthermore, E2F3/RB1 cases rarely evolve from FGFR3/CCND1 tumors as very few of the former harbor *FGFR3* or *PIK3CA* mutations, and in particular, do not harbor homozygous deletions of *CDKN2A*. Thus, our data suggest the presence of two complementary pathways operating in UC, one characterized by FGFR3/CCND1 and a second by the E2F3/RB1 alterations. We also demonstrated that E2F3/RB1 altered tumors show overexpression of *CDKN2A*, which, at first hand, may be surprising. However, these tumors are most likely less sensitive to *CDKN2A* levels due to the aberrant E2F3/RB1 expression, and may thus tolerate a stronger p16 induced senescence signal.

We finally identified a gene expression subgroup of high grade tumors which was relatively heterogeneous with respect to the chromosomal alterations that defined the above genomic circuits. This group of tumors showed no distinct pattern of genomic alterations, except for enrichment of CCND1 amplifications. Intriguingly, this group had the worst prognosis. A clue to the nature of these tumors was obtained by gene expression analysis; increased expression of genes associated with keratinization was observed, thus suggesting signs of squamous metaplasia. Importantly, these findings were corroborated by a subsequent pathological reevaluation. Hence, these tumors may have acquired additional genomic and/or epigenetic hits that promote a squamous like phenotype. However, the relatively low frequency of these tumors implies that a larger sample material is needed to describe this circuit further.

## Materials and Methods

### Sample acquisition and mutation analyses

Urothelial carcinomas were collected by cold-cup biopsies from the exophytic region of the bladder tumor in 146 patients undergoing transurethral resection at the Department of Urology, Skåne University Hospital, Sweden. Written informed consent was obtained from all patients, and the study was approved by the Local Ethical Committee of Lund University. Follow up data was available for 145 of the patients (median follow-up time 56 months). RNA was isolated using Trizol (Invitrogen) and purified on Qiagen RNeasy columns (Qiagen). RNA sample integrity was assessed on an Agilent 2100 Bioanalyzer (Agilent technologies). Genomic DNA was extracted using the DNeasy Tissue kit protocol (Qiagen) or using the organic phase of the Trizol lysate according to the manufacturers instructions. Mutation status for *FGFR3*, *PIK3CA*, *KRAS*, *HRAS*, *NRAS*, *CDKN2A*, and *TP53* were determined by direct sequencing as described in Sjödahl et al. [11] (Table S1). Patient data is summarized in Table 1 and detailed sample information and mutation data are given in Table S2.

**Table 1:** Summary of patient material.

**Tumor Stage (n)**	
Ta	49
T1	53
≥T2	42
Tx	1
Tis	1
**Tumor Grade (n)**	
G1	19
G2	46
G3	81
**Median age, years (range)**	70 (38–90)
**Gender (n)**	
Female	32
Male	114
**Mutation frequencies (%)**	
FGFR3	38.4
PIK3CA	16.7
RAS	6.2
TP53	39.7
CDKN2A	1.9

### Genomic profiling and gene expression analyses

Genomic DNA was hybridized to 32K BAC arrays produced at the SCIBLU Genomics Centre (http://www-lth.se/sciblu) at Lund University. Genomic profiles for 36 samples were included in Heidenblad et al. [9]. Hybridization, data normalization, and segmentation were performed as described previously [9], [15].

Gene expression profiling was performed using Illumina humanHT12v3 BeadChips (Illumina Inc) on 131 samples from which total RNA was available. Samples were randomly distributed into two separate labeling batches. To correct for labeling specific biases, raw intensity values were subjected to a labeling specific transformation step, in which for each probe on the array, the geometric mean of signal intensities for samples in the second batch was scaled to the geometric mean of samples on the first labeling batch. Intensity values were then background corrected, negative values were capped to zero, and an intensity constant of 30 was added to all probe intensities. Data were normalized using a quantile normalization algorithm as implemented in BASE [23], log2 transformed, and variance filtered (SD>0.25). Raw data files as well as filtered and normalized data matrixes are supplied on the NCBI GEO web site (http://www.ncbi.nlm.nih.gov/geo/) through accession number GSE32549.

### Identification of copy number alterations

Genomic gains and deletions were defined from segmented log2 data using sample adaptive thresholds (SAT) on 250 kb smoothed data [24]. High-level focal genomic amplifications (FGAs) and homozygous deletions (HDs) were defined as segmented log2 ratios ≥0.8 or ≤−0.8, respectively, and only segments longer than three consecutive BAC probes were considered. FGAs less than 1 Mbp apart were considered as one single amplicon when calculating FGA frequencies. Recurrent FGAs were defined as genomic segments for which at least three samples were amplified, recurrent HDs as regions for which at least two samples were altered, and minimal regions of deletions (MRD) were defined by a minimum of 15 cases, as outlined in Figure S1. Recurrent aberrations were crosschecked against known copy-number variants (CNVs) available through the Copy Number Variation Project (http://www.sanger.ac.uk/humgen/cnv/data/cnv_data/) and the Database of Genomic Variants (http://projects.tcag.ca/variation/), and regions with high CNV overlap were removed from further analyses. Large recurrent chromosome arm gains and losses were defined as cases in which >50% of BAC probes for a chromosome arm were above or below the respective SAT level in more than 10% of the samples.

### Immunohistochemistry

Tissue microarray (TMA) blocks were constructed from 1.0 mm punches of formalin fixed paraffin embedded tissue specimens. All cores were selected by a pathologist to contain sufficient tumor material for scoring of tumor cells. 119 tumors were represented on the TMAs. Two cores per sample were included, except for 3 samples that were represented by a single core. Monoclonal antibodies against FGFR3 (clone C51F2, Cell Signaling), CCND1 (clone SP4, Dako), RB1 (clone 4H1, Cell signaling), E2F3 (clone 3E2F04, Labvision), p16 (clone G175-405, BD biosciences) were used. TMA sections were pretreated using Dako PT Link pH 9, stained using Dako Autostainer, and visualized using Dako EnVision ^™^FLEX K8010 (Dako). As negative control, the primary antibody was omitted. Staining intensities were assigned a score of 0–3 and the fraction of positive tumor cells/nuclei was estimated using 10% intervals (0%–100%). The tumor cell protein score was calculated by multiplying the intensity with the fraction of positive tumor nuclei. For samples represented by two cores, the mean score was used.

### Statistical analyses

Statistical analyses were performed using the R software (http://www.r-project.org). Multidimensional scaling (MDS) was performed using Jaccard distances based on the presence or absence of recurrent alterations. FGA regions present in limited number of samples (<5%) were not included in these analyses. Hierarchical Cluster Analyses (HCA) was performed using Pearson correlation and Ward's method for agglomeration. Survival curves were compared using Kaplan-Meier estimates and disease specific survival (DSS) was used as end point. Differential gene expression between sample groups was determined using the limma R package [25] and Bonferroni corrected p-values<0.05 were considered significant.

## Supporting Information

Figure S1 **Schematic illustration on how recurrent focal genomic amplifications (FGAs), homozygous deletions (HDs), and minimal regions of deletion (MRDs) were defined from segmented genomic profiles of 146 tumors.** Orange horizontal bars above the ideogram represent samples with segments of amplification (relative copy number log2>0.8). Blue bars below the ideogram illustrate samples with deletions (log2<SAT) and dark blue bars represents segments of homozygous deletion (log2<−0.8). For recurrent FGA regions a minimum of 3 samples had to show amplification. For MRDs the limit was set to 15 cases and for HDs a minimum of 2 cases. First, core regions were defined representing local peak maxima (black vertical lines), i.e. the minimal shared chromosomal region for amplifications, deletions, and homozygous deletions, respectively (indicated by black arrowheads). To allow for uncertain measurements due to technical noise, the boundaries for each core region was increased to include two cases less altered than the maximum number of altered cases for that region (red arrowheads and red vertical lines). For HDs this limit was set to one sample less than the maximum number. In cases of overlap between MRD and HD regions, the region defined by HD boundaries was used. Recurrent aberrations were crosschecked against known copy-number variants (CNVs) available through the Copy Number Variation Project and the Database of Genomic Variants, and regions with high CNV overlap were removed from further analyses. In some instances, regions of deletions were too large to accurately define MRDs. For example, most samples carried whole or near whole arm deletions of 5q and the only MRD peak that could be identified corresponded to a known CNV (visible in Fig. 1B). Therefore no MRDs were defined on this chromosome arm. Instead we used a measure to include large and recurrent deletions of chromosome arms. The large gains and losses were defined as cases in which >50% of BAC probes for a chromosome arm were above or below the respective SAT level and were defined as recurrent present in more than 10% of the samples.
(TIF)

Figure S2 **DNA copy number frequency plot of gains (red) and losses (blue).** Tumors are stratified according to **A**) grade and **B**) stage, respectively. The presence of gains and deletions were defined from the segmented log2 data using sample adaptive thresholds (SAT) on 250 kb smoothed data [24].
(TIF)

Figure S3 **Hierarchical cluster analysis (HCA) based on global gene expression.** The analysis was performed on a variance filtered expression matrix (SD>0.25) Representing relative transcript levels for 18997 reporters in 131 tumors.
(TIF)

Figure S4 **Gene expression rank plots.** Relative gene expression (y-axis) obtained from global gene expression analysis of 131. Samples are ordered from the lowest expressing sample to the highest expressing sample (x-axis). **A**) *MDM2* gene expression. Samples with *TP53* mutation or *MDM2* amplifications are indicated with red lines and red solid circles, respectively. Samples with expression above the horizontal dashed line were considered to overexpress *MDM2*. **B**) Rank plots used to define categorized variables for *FGFR3*, *CCND1*, *RAF1*, *CDKN2A*, *PTEN*, and *RB1* gene expression levels. For *CCND1*, *RAF1*, and *CDKN2A*, samples with elevated expression relative to the dashed red horizontal line were considered *CCND1* ^high^ *RAF1* ^high^, and *CDKN2A* ^high^, respectively. For *PTEN* and *RB1*, samples with lower expression than indicated by the dashed line were defined as low expressers (*PTEN^low^* and *RB1^low^*, respectively). *FGFR3* expression was divided into *FGFR3^high^* and *FGFR3^low^* expression (above or below the two dashed lines, respectively).
(TIF)

Figure S5 **Genomic alterations at three chromosome arms for samples in the HC5, HC3c, and HC2 subgroups.** The *RB1*, *E2F3*, and *CDKN2A* loci are indicated by vertical lines. Three HC5 samples harbor intrachromosomal breakpoints surrounding the *RB1* locus (arrowheads). Segments of genomic imbalances are color-coded as follows: amplifications (red), gains (orange), no alteration (gray), deletions (blue), and homozygous deletions (dark blue).
(TIF)

Figure S6 **Relative mRNA transcript levels and protein expression of the CDKN2A gene.** **A**) Rank plot of *CDKN2A* gene expression. Samples with 6p22 amplifications and/or *RB1* deletions are colored in red. Samples with homozygous deletions at the *CDKN2A* locus are indicated with blue lines. **B**) Relative p16 protein expression in normal urothelium (n = 3), as well as for tumors with no detected *E2F3* amplification or *RB1* deletion (n = 84) and tumors with *E2F3* amplification and/or *RB1* deletion (n = 35), as assessed using TMA. Protein expression was calculated by multiplying the intensity with the fraction of positive tumor cell nuclei. For samples represented by two cores, the mean score was used. **C–E**) Representative IHC stainings of p16 protein expression in **C**) normal urothelium **D**) tumors without amplification of *E2F3* or *RB1* deletions, and **E**) tumors with *E2F3* amplification.
(TIF)

Table S1 **List of recurrent regions of focal genomic amplification (FGA), deletions (MRD) and homozygous deletions (HD).** For each region, the corresponding cytoband, genomic position, and start and end BAC reporter is given.
(XLSX)

Table S2 **Detailed sample information for the 146 tumors included in the study.** The TNM 2002 and WHO 1999 classification systems were used for tumor staging and grading, respectively. DOD, dead of disease. NA, data not available.
(XLS)

## References

[1] Richter J, Jiang F, Gorog JP, Sartorius G, Egenter C (1997). Marked genetic differences between stage pTa and stage pT1 papillary bladder cancer detected by comparative genomic hybridization.. Cancer research.

[2] Hoglund M, Sall T, Heim S, Mitelman F, Mandahl N (2001). Identification of cytogenetic subgroups and karyotypic pathways in transitional cell carcinoma.. Cancer research.

[3] Fadl-Elmula I (2005). Chromosomal changes in uroepithelial carcinomas.. Cell & chromosome.

[4] Richter J, Beffa L, Wagner U, Schraml P, Gasser TC (1998). Patterns of chromosomal imbalances in advanced urinary bladder cancer detected by comparative genomic hybridization.. The American journal of pathology.

[5] Majewski T, Lee S, Jeong J, Yoon DS, Kram A (2008). Understanding the development of human bladder cancer by using a whole-organ genomic mapping strategy.. Laboratory investigation; a journal of technical methods and pathology.

[6] Veltman JA, Fridlyand J, Pejavar S, Olshen AB, Korkola JE (2003). Array-based comparative genomic hybridization for genome-wide screening of DNA copy number in bladder tumors.. Cancer research.

[7] Blaveri E, Brewer JL, Roydasgupta R, Fridlyand J, DeVries S (2005). Bladder cancer stage and outcome by array-based comparative genomic hybridization.. Clinical cancer research: an official journal of the American Association for Cancer Research.

[8] Hurst CD, Fiegler H, Carr P, Williams S, Carter NP (2004). High-resolution analysis of genomic copy number alterations in bladder cancer by microarray-based comparative genomic hybridization.. Oncogene.

[9] Heidenblad M, Lindgren D, Jonson T, Liedberg F, Veerla S (2008). Tiling resolution array CGH and high density expression profiling of urothelial carcinomas delineate genomic amplicons and candidate target genes specific for advanced tumors.. BMC medical genomics.

[10] Gui Y, Guo G, Huang Y, Hu X, Tang A (2011). Frequent mutations of chromatin remodeling genes in transitional cell carcinoma of the bladder.. Nature genetics.

[11] Sjodahl G, Lauss M, Gudjonsson S, Liedberg F, Hallden C (2011). A systematic study of gene mutations in urothelial carcinoma; inactivating mutations in TSC2 and PIK3R1.. PloS one.

[12] Billerey C, Chopin D, Aubriot-Lorton MH, Ricol D, Gil Diez de Medina S (2001). Frequent FGFR3 mutations in papillary non-invasive bladder (pTa) tumors.. The American journal of pathology.

[13] Wu XR (2005). Urothelial tumorigenesis: a tale of divergent pathways.. Nature reviews Cancer.

[14] Goebell PJ, Knowles MA (2010). Bladder cancer or bladder cancers? Genetically distinct malignant conditions of the urothelium.. Urologic oncology.

[15] Lindgren D, Frigyesi A, Gudjonsson S, Sjodahl G, Hallden C (2010). Combined gene expression and genomic profiling define two intrinsic molecular subtypes of urothelial carcinoma and gene signatures for molecular grading and outcome.. Cancer research.

[16] Schvartzman JM, Sotillo R, Benezra R (2010). Mitotic chromosomal instability and cancer: mouse modelling of the human disease.. Nature reviews Cancer.

[17] Bild AH, Yao G, Chang JT, Wang Q, Potti A (2006). Oncogenic pathway signatures in human cancers as a guide to targeted therapies.. Nature.

[18] Ceol CJ, Houvras Y, Jane-Valbuena J, Bilodeau S, Orlando DA (2011). The histone methyltransferase SETDB1 is recurrently amplified in melanoma and accelerates its onset.. Nature.

[19] Williams SV, Sibley KD, Davies AM, Nishiyama H, Hornigold N (2002). Molecular genetic analysis of chromosome 9 candidate tumor-suppressor loci in bladder cancer cell lines.. Genes, chromosomes & cancer.

[20] Haigis KM, Sweet-Cordero A (2011). New insights into oncogenic stress.. Nature genetics.

[21] Hurst CD, Tomlinson DC, Williams SV, Platt FM, Knowles MA (2008). Inactivation of the Rb pathway and overexpression of both isoforms of E2F3 are obligate events in bladder tumours with 6p22 amplification.. Oncogene.

[22] Puzio-Kuter AM, Castillo-Martin M, Kinkade CW, Wang X, Shen TH (2009). Inactivation of p53 and Pten promotes invasive bladder cancer.. Genes & development.

[23] Vallon-Christersson J, Nordborg N, Svensson M, Hakkinen J (2009). BASE–2nd generation software for microarray data management and analysis.. BMC bioinformatics.

[24] Staaf J, Jonsson G, Ringner M, Vallon-Christersson J (2007). Normalization of array-CGH data: influence of copy number imbalances.. BMC genomics.

[25] Smyth GK, Michaud J, Scott HS (2005). Use of within-array replicate spots for assessing differential expression in microarray experiments.. Bioinformatics.

