# Supplementary figures and images for: Integrated Genomic and Gene Expression Profiling Identifies Two Major Genomic Circuits in Urothelial Carcinoma
Source: PLoS One. 2012 Jun 7;7(6):e38863. doi: 10.1371/journal.pone.0038863 (PMC3369837; doi:10.1371/journal.pone.0038863)

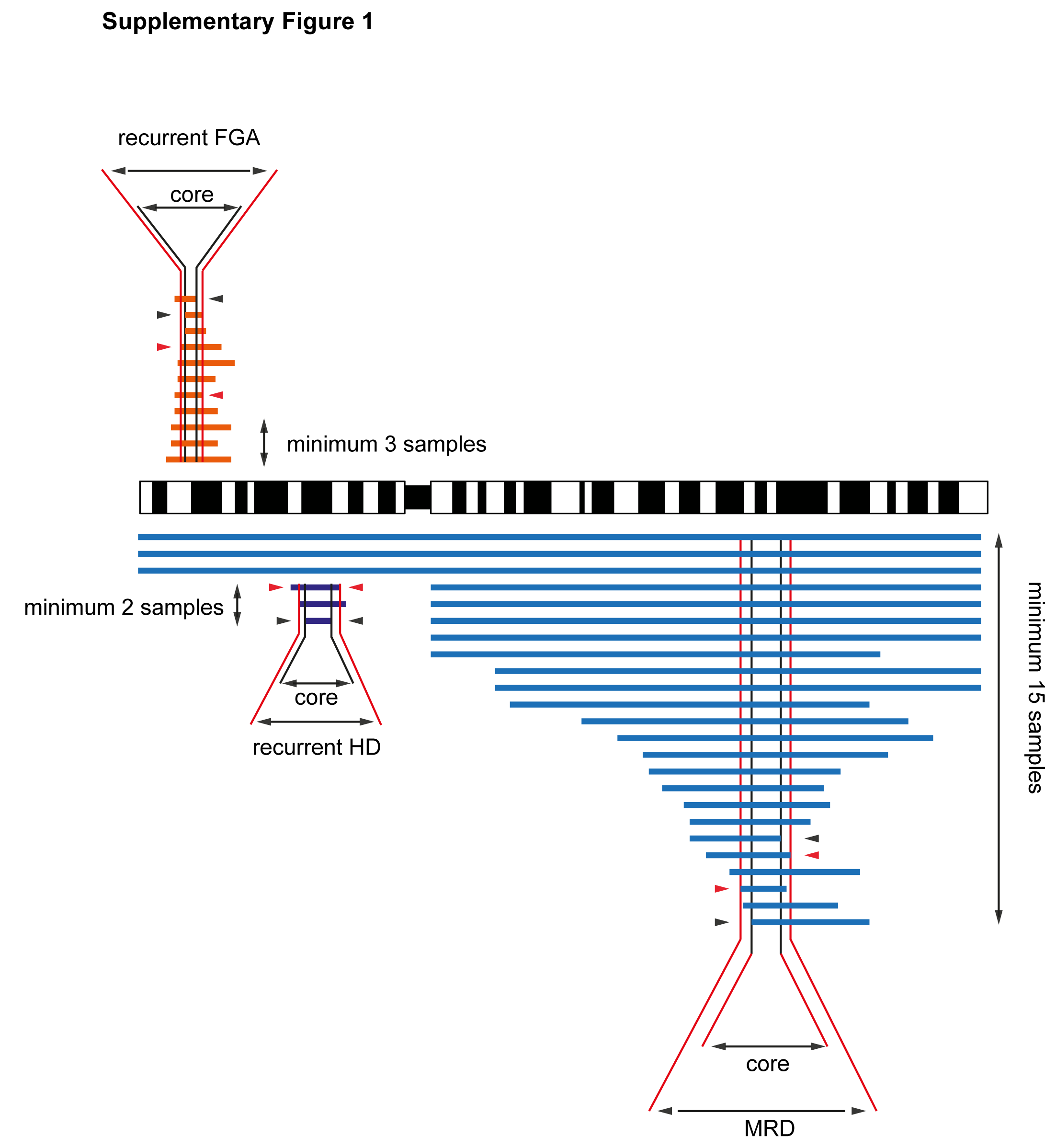

Supplement: Figure S1 — Schematic illustration on how recurrent focal genomic amplifications (FGAs), homozygous deletions (HDs), and minimal regions of deletion (MRDs) were defined from segmented genomic profiles of 146 tumors. Orange horizontal bars above the ideogram represent samples with segments of amplification (relative copy number log2>0.8). Blue bars below the ideogram illustrate samples with deletions (log2<SAT) and dark blue bars represents segments of homozygous deletion (log2<−0.8). For recurrent FGA regions a minimum of 3 samples had to show amplification. For MRDs the limit was set to 15 cases and for HDs a minimum of 2 cases. First, core regions were defined representing local peak maxima (black vertical lines), i.e. the minimal shared chromosomal region for amplifications, deletions, and homozygous deletions, respectively (indicated by black arrowheads). To allow for uncertain measurements due to technical noise, the boundaries for each core region was increased to include two cases less altered than the maximum number of altered cases for that region (red arrowheads and red vertical lines). For HDs this limit was set to one sample less than the maximum number. In cases of overlap between MRD and HD regions, the region defined by HD boundaries was used. Recurrent aberrations were crosschecked against known copy-number variants (CNVs) available through the Copy Number Variation Project and the Database of Genomic Variants, and regions with high CNV overlap were removed from further analyses. In some instances, regions of deletions were too large to accurately define MRDs. For example, most samples carried whole or near whole arm deletions of 5q and the only MRD peak that could be identified corresponded to a known CNV (visible in Fig. 1B). Therefore no MRDs were defined on this chromosome arm. Instead we used a measure to include large and recurrent deletions of chromosome arms. The large gains and losses were defined as cases in which >50% of BAC probes for a chromosome ar [file pone.0038863.s001.tif]

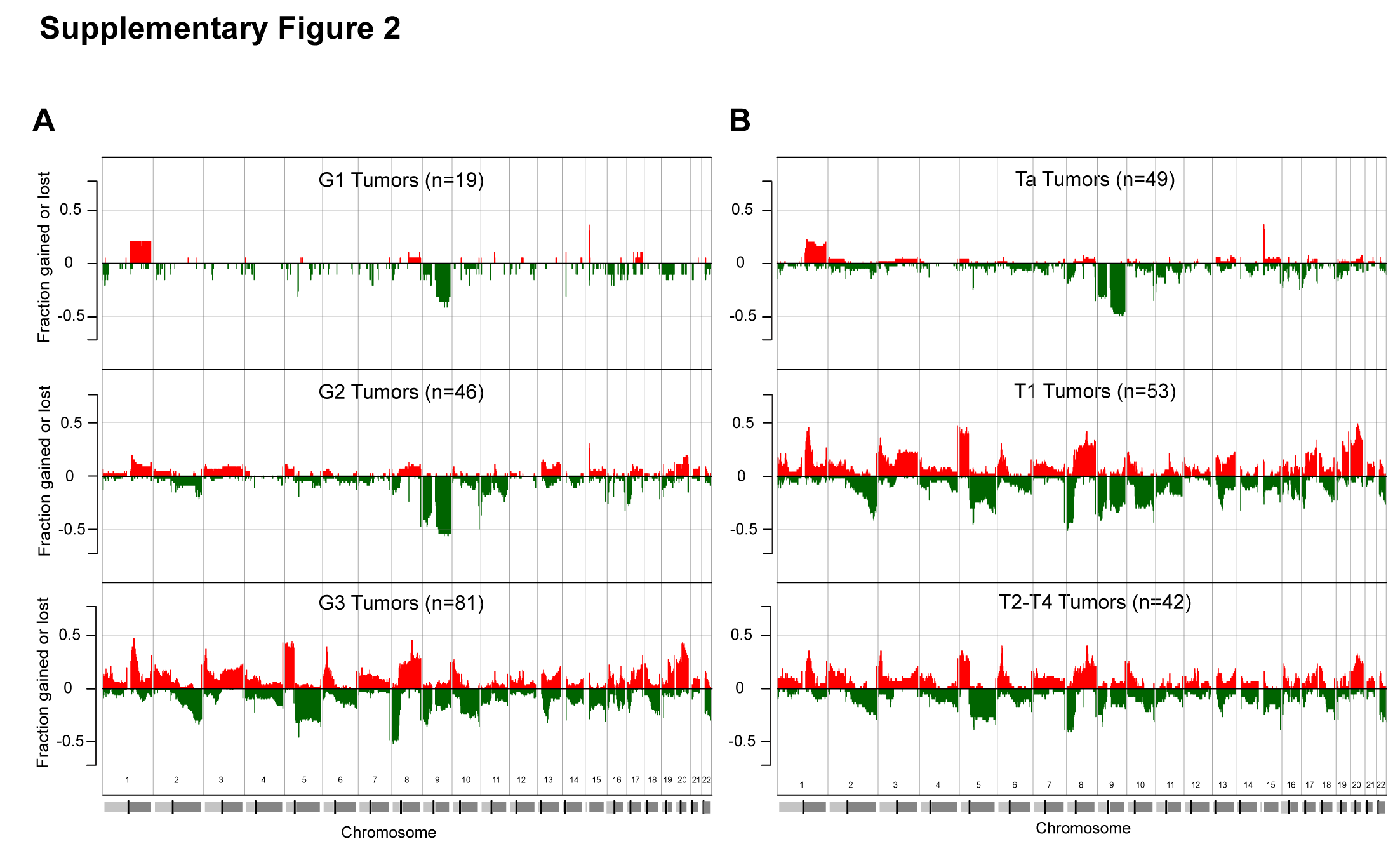

Supplement: Figure S2 — DNA copy number frequency plot of gains (red) and losses (blue). Tumors are stratified according to A) grade and B) stage, respectively. The presence of gains and deletions were defined from the segmented log2 data using sample adaptive thresholds (SAT) on 250 kb smoothed data [24]. (TIF) [file pone.0038863.s002.tif]

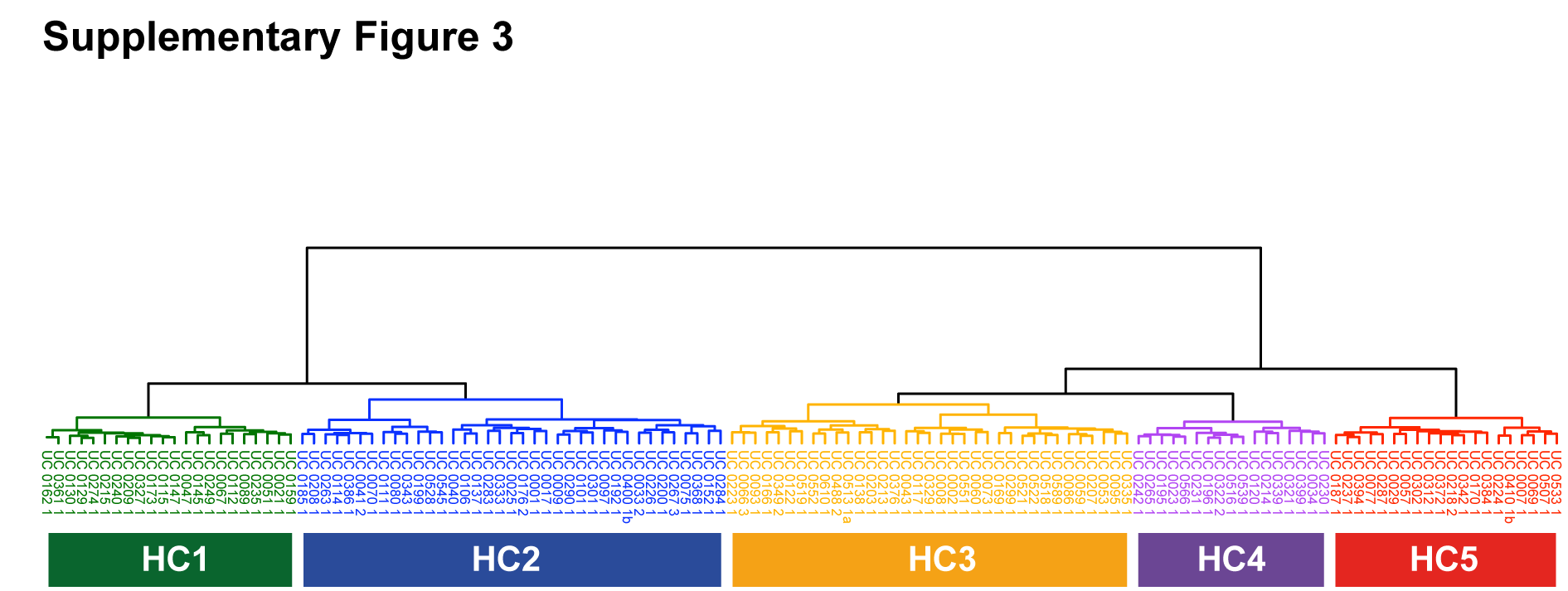

Supplement: Figure S3 — Hierarchical cluster analysis (HCA) based on global gene expression. The analysis was performed on a variance filtered expression matrix (SD>0.25) Representing relative transcript levels for 18997 reporters in 131 tumors. (TIF) [file pone.0038863.s003.tif]

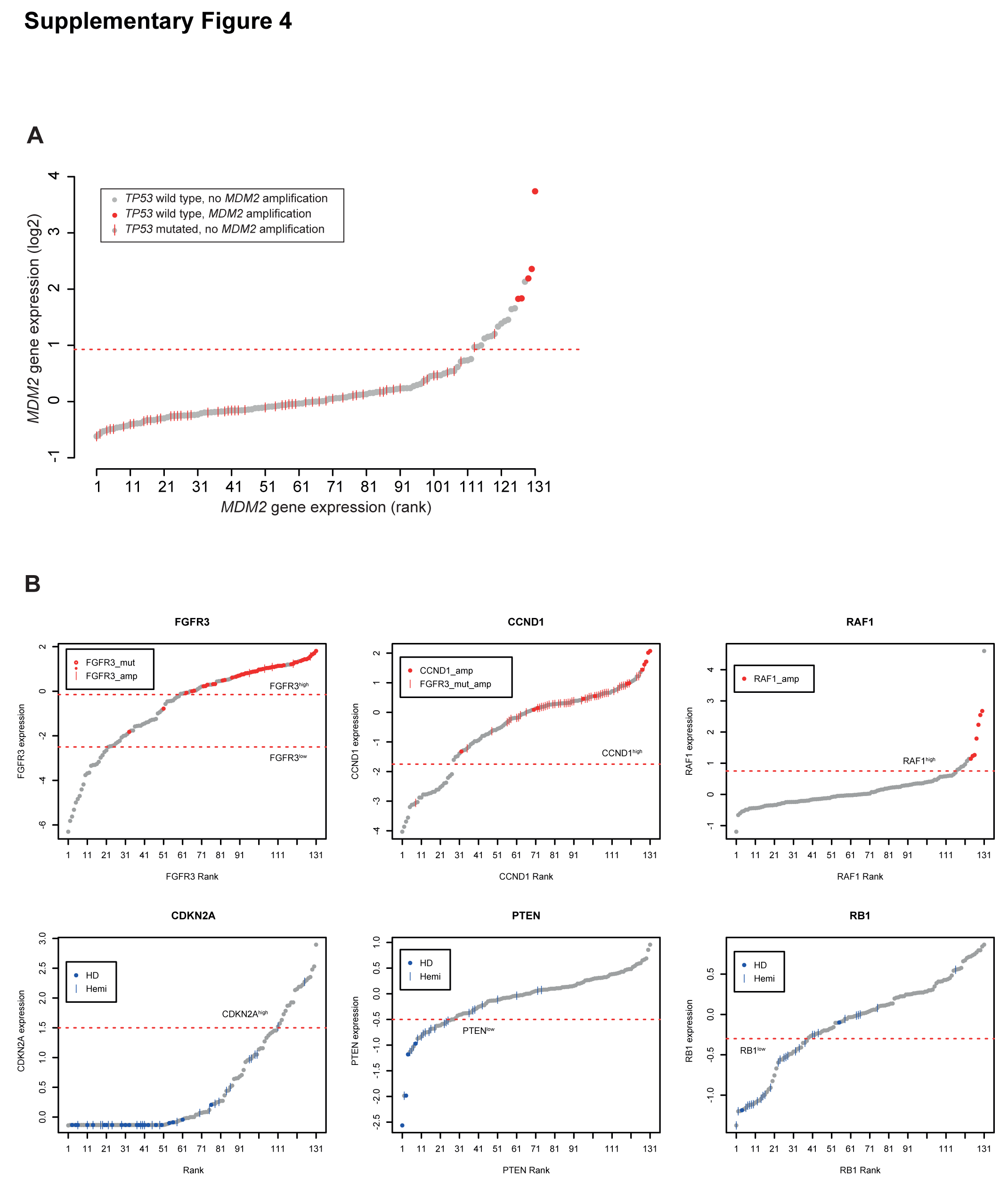

Supplement: Figure S4 — Gene expression rank plots. Relative gene expression (y-axis) obtained from global gene expression analysis of 131. Samples are ordered from the lowest expressing sample to the highest expressing sample (x-axis). A) MDM2 gene expression. Samples with TP53 mutation or MDM2 amplifications are indicated with red lines and red solid circles, respectively. Samples with expression above the horizontal dashed line were considered to overexpress MDM2. B) Rank plots used to define categorized variables for FGFR3, CCND1, RAF1, CDKN2A, PTEN, and RB1 gene expression levels. For CCND1, RAF1, and CDKN2A, samples with elevated expression relative to the dashed red horizontal line were considered CCND1 high RAF1 high, and CDKN2A high, respectively. For PTEN and RB1, samples with lower expression than indicated by the dashed line were defined as low expressers (PTENlow and RB1low, respectively). FGFR3 expression was divided into FGFR3high and FGFR3low expression (above or below the two dashed lines, respectively). (TIF) [file pone.0038863.s004.tif]

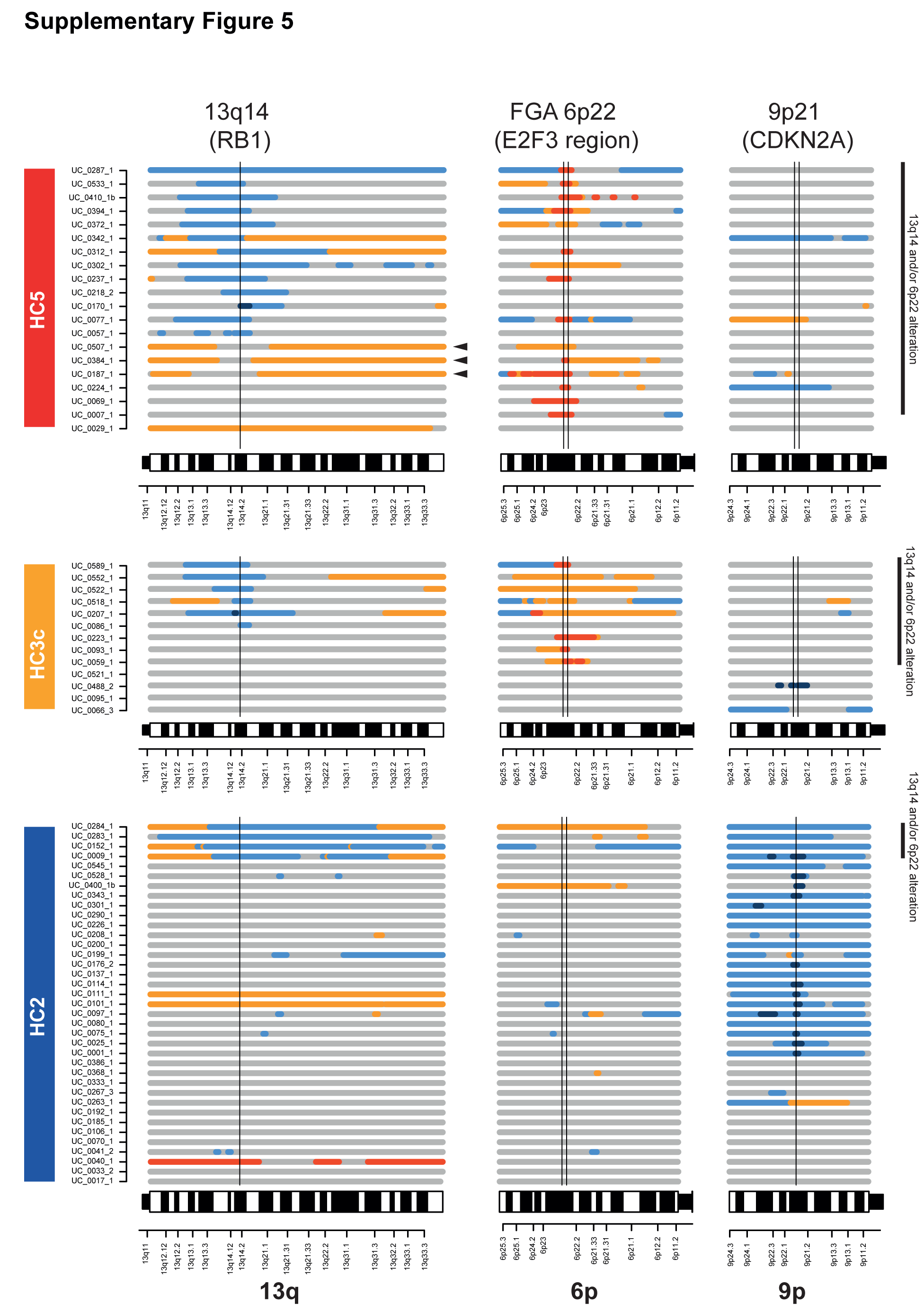

Supplement: Figure S5 — Genomic alterations at three chromosome arms for samples in the HC5, HC3c, and HC2 subgroups. The RB1, E2F3, and CDKN2A loci are indicated by vertical lines. Three HC5 samples harbor intrachromosomal breakpoints surrounding the RB1 locus (arrowheads). Segments of genomic imbalances are color-coded as follows: amplifications (red), gains (orange), no alteration (gray), deletions (blue), and homozygous deletions (dark blue). (TIF) [file pone.0038863.s005.tif]

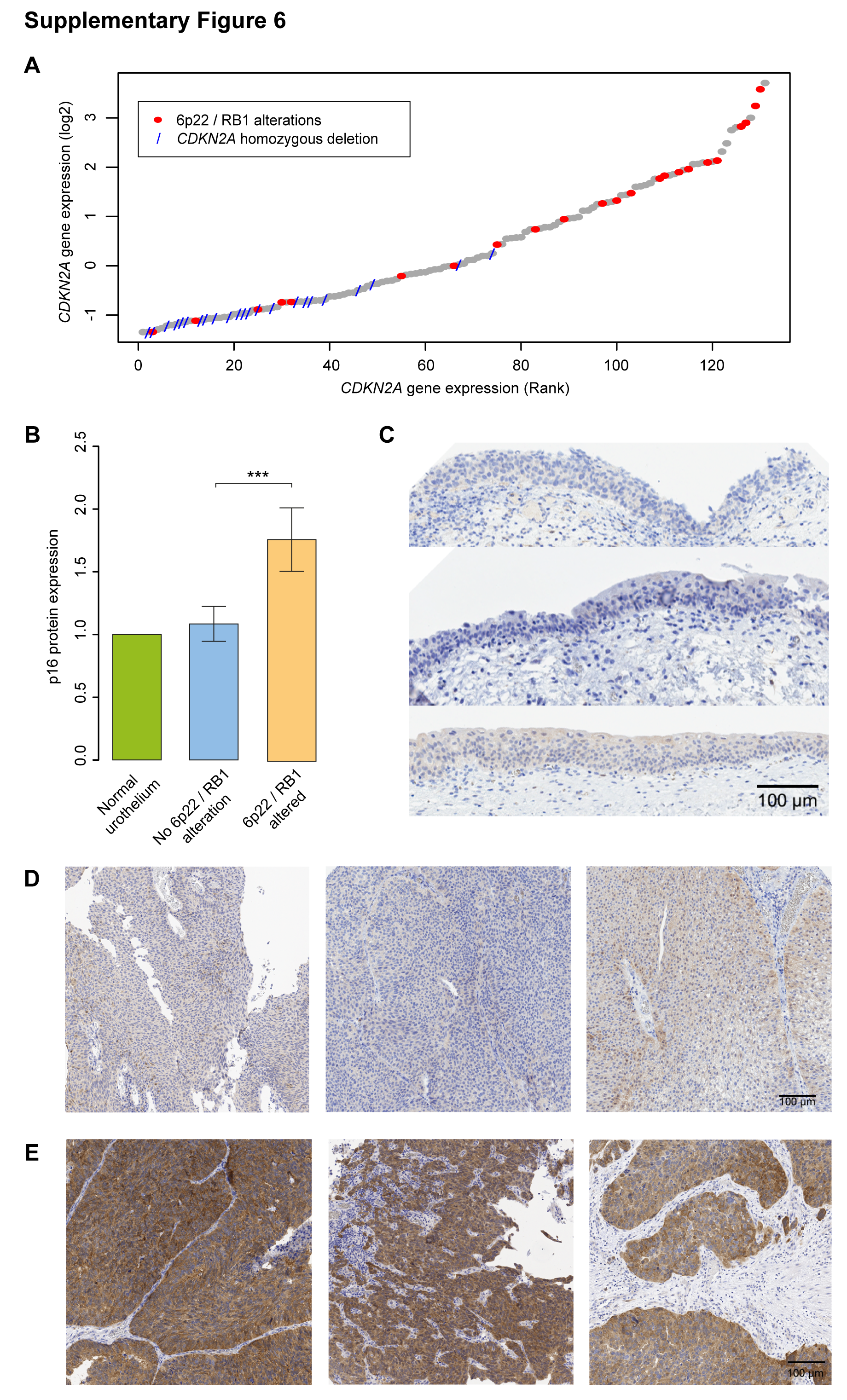

Supplement: Figure S6 — Relative mRNA transcript levels and protein expression of the CDKN2A gene. A) Rank plot of CDKN2A gene expression. Samples with 6p22 amplifications and/or RB1 deletions are colored in red. Samples with homozygous deletions at the CDKN2A locus are indicated with blue lines. B) Relative p16 protein expression in normal urothelium (n = 3), as well as for tumors with no detected E2F3 amplification or RB1 deletion (n = 84) and tumors with E2F3 amplification and/or RB1 deletion (n = 35), as assessed using TMA. Protein expression was calculated by multiplying the intensity with the fraction of positive tumor cell nuclei. For samples represented by two cores, the mean score was used. C–E) Representative IHC stainings of p16 protein expression in C) normal urothelium D) tumors without amplification of E2F3 or RB1 deletions, and E) tumors with E2F3 amplification. (TIF) [file pone.0038863.s006.tif]
